# Improving Genomic Selection With Quantitative Trait Loci and Nonadditive Effects Revealed by Empirical Evidence in Maize

**DOI:** 10.3389/fpls.2019.01129

**Published:** 2019-09-18

**Authors:** Xiaogang Liu, Hongwu Wang, Xiaojiao Hu, Kun Li, Zhifang Liu, Yujin Wu, Changling Huang

**Affiliations:** Institute of Crop Science, National Key Facility of Crop Gene Resources and Genetic Improvement, Chinese Academy of Agricultural Sciences, Beijing, China

**Keywords:** maize, genomic selection, association and linkage mapping, trait-relevant marker, nonadditive effect, population structure

## Abstract

Genomic selection (GS), a tool developed for molecular breeding, is used by plant breeders to improve breeding efficacy by shortening the breeding cycle and to facilitate the selection of candidate lines for creating hybrids without phenotyping in various environments. Association and linkage mapping have been widely used to explore and detect candidate genes in order to understand the genetic mechanisms of quantitative traits. In the current study, phenotypic and genotypic data from three experimental populations, including data on six agronomic traits (e.g., plant height, ear height, ear length, ear diameter, grain yield per plant, and hundred-kernel weight), were used to evaluate the effect of trait-relevant markers (TRMs) on prediction accuracy estimation. Integrating information from mapping into a statistical model can efficiently improve prediction performance compared with using stochastically selected markers to perform GS. The prediction accuracy can reach plateau when a total of 500–1,000 TRMs are utilized in GS. The prediction accuracy can be significantly enhanced by including nonadditive effects and TRMs in the GS model when genotypic data with high proportions of heterozygous alleles and complex agronomic traits with high proportion of nonadditive variancein phenotypic variance are used to perform GS. In addition, taking information on population structure into account can slightly improve prediction performance when the genetic relationship between the training and testing sets is influenced by population stratification due to different allele frequencies. In conclusion, GS is a useful approach for prescreening candidate lines, and the empirical evidence provided by the current study for TRMs and nonadditive effects can inform plant breeding and in turn contribute to the improvement of selection efficiency in practical GS-assisted breeding programs.

## Introduction

Genomic selection (GS) has been widely implemented to powerfully assist in modern animal and plant breeding (Xu et al., 2016; Nirea and Meuwissen, 2017; Raoul et al., 2017; Zhang et al., 2017b; Robledo et al., 2018; Brandariz and Bernardo, 2019; Rezende et al., 2019; Sarinelli et al., 2019; Yuan et al., 2019) and has the ability to utilize genome-wide markers (e.g., single nucleotide polymorphisms, SNPs) to accelerate the selection procedure, with the assumption that each marker is associated with minor genetic effects originally proposed by Meuwissen in 2001 in a discussion of several statistical models (Meuwissen et al., 2001). Several factors, including marker density, population size, genetic relationships, statistical models, and breeding platforms, have an impact on the estimation of marker effects that are generally recognized as random effects in the models and thus can impact prediction accuracy (Crossa et al., 2017; Hickey et al., 2017; Schopp et al., 2017; Zhang et al., 2017a; Liu et al., 2018; Wang et al., 2018; Zhang et al., 2019). In general, the estimated effects of each marker follow a normal distribution with the same or different variances under *a priori* assumptions in a model based on penalized (e.g., ridge regression best linear unbiased prediction, RR-BLUP; genomic BLUP, GBLUP) (Whittaker et al., 2000; VanRaden, 2008; Endelman, 2011) or Bayesian approaches (e.g., BayesA, BayesB, and BayesC) (Meuwissen et al., 2001; Habier et al., 2011). However, some polymorphic markers should be virtually evaluated as having stronger genetic effects and other markers are estimated to have weaker genetic effects because these markers do not have biological functions for the target agronomic traits (Bernardo, 2014; Arruda et al., 2016; Boeven et al., 2016; Spindel et al., 2016; Bian and Holland, 2017). In fact, conventional approaches in quantitative genetics, such as genome-wide association studies (GWASs) and quantitative trait locus (QTL) mapping, can efficiently dissect the genetic architecture of target traits and aid in the exploration of candidate genes for the development of functional markers (Li et al., 2013; Wang et al., 2016; Xiao et al., 2016; Pan et al., 2017; Zhang et al., 2017c). In addition, these functional or trait-relevant markers (TRMs) can explain a large fraction of the genetic variance, which may improve the predictive ability of GS models in plant and animal breeding (Su et al., 2014; Zhang et al., 2014; Ober et al., 2015; Zhang et al., 2015; Arruda et al., 2016; Boeven et al., 2016; Bian and Holland, 2017; Kemper et al., 2018). Several previous studies discussed the advantages of combining GWASs and GS, which usually take TRMs as fixed effects in statistical models (Spindel et al., 2016; Herter et al., 2019; Rice and Lipka, 2019).

With the improvement of statistical models, predication accuracy has increased due to the consideration of supplementary effects, such as nonadditive, fixed, and genotype-by-environment interaction effects (Lopez-Cruz et al., 2015; Zhao et al., 2015; Boeven et al., 2016; Lado et al., 2016; Alves et al., 2019; Herter et al., 2019). Integrating nonadditive effects into statistical models can significantly improve prediction accuracy when the nonadditive variance possesses a relatively large proportion of genetic variance (Su et al., 2012; Azevedo et al., 2015; Liu et al., 2018; Varona et al., 2018). Furthermore, models including nonadditive effects have been widely applied to evaluate genomic estimated breeding values (GEBVs) of individuals in the process of hybrid selection (Xu et al., 2014; Miedaner et al., 2017; Fristche-Neto et al., 2018; Werner et al., 2018). Compared to a model that considers only additive effects, the improved model can explain more fraction of genetic variance, which can further explore and dissect genetic effects of genomic markers (Da et al., 2014; Bouvet et al., 2016; Morais Júnior et al., 2017; Alves et al., 2019). On the other hand, several previous studies evaluated the effects of population structure with an experimental design in which the genetic distance changed from lessrelevant to morerelevant between training and testing sets (Guo et al., 2014; Isidro et al., 2015; Zhang et al., 2017a; Rio et al., 2019). In fact, population structure that is mainly attributed to different allele frequencies between groups can further impact the construction of genomic relationships and estimation of GEBVs and then affect the predictive ability of GS models (Liu et al., 2018). Information on population structure can be explicitly considered as fixed effects in the models, but significant enhancement of prediction accuracy does not occur (Rio et al., 2019). Furthermore, population structure has less effects on prediction performance when GS is performed with only a specific group or within a subpopulation (Guo et al., 2014). For the description of population structure, principal component analysis (PCA) is an efficient approach based on genomic information (Price et al., 2006). Generally, a PC matrix is used to explicitly illustrate population stratification in GWASs (Price et al., 2006; Huang et al., 2010; Slavov et al., 2014; Huang et al., 2015; Chang et al., 2018) but is rarely applied to correct the effect of population structure in GS models. Hence, taking information on population structure into account will have the benefit of adjusting the bias of estimated marker effects generated by population stratification, likely making great progress in improving prediction performance.

With respect to the application of TRMs in GS, many studies have argued in favor of including TRMs as fixed effects in statistical models to enhance prediction accuracy. However, few reports directly integrating TRMs into GS models have been published (Ober et al., 2015; Yuan et al., 2019). In this study, we primarily aimed to discuss the effect of TRMs and the integration of TRMs and other effects in order to provide recommendations that can assist plant breeders in the design of GS-assisted breeding programs. Phenotypic and genotypic data from three experimental populations, including one natural and two biparental populations, which included six agronomic traits and a 55 K SNP array, were collected. Our objectives were to (1) assess the accuracy and quality of association and linkage mapping, (2) evaluate the effect of TRMs identified by association and linkage mapping performed using data from training sets, (3) investigate the degree to which nonadditive effects in combination with TRMs influence prediction accuracy, and (4) integrate information on population structure into a mixed model as a fixed effect to improve predictive ability. Finally, these results were used to provide pertinent advice for improving GS schemes in commercial breeding programs.

## Materials and Methods

### Plant Materials and Experimental Management

The plant materials were described in detail by Liu et al. (2018). In total, three experimental populations, which included one natural and two biparental populations, were used in this study.More specifically, a total of 435 elite maize inbred lines were used to construct the natural population, and the two biparental populations, which were derived from one single-cross maize hybrid with the elite inbred lines Zheng58 and HD568 as parents that included in the natural population, consisted of 212 recombinant inbred lines (RILs) and 304 F_2:3_ families, respectively. The natural population was grown in Henan Province in 2014 and 2015. The two biparental populations were evaluated in the same location in 2015 and 2016. A field trial with a randomized incomplete block design was performed with two replicates. Six yield-related agronomic traits constituted the phenotypic data: plant height (PH, cm), ear height (EH, cm), ear length (EL, mm), ear diameter (ED, mm), grain yield per plant (GYP, kg), and hundred-kernel weight (HKW, g). Furthermore, phenotypic values of HKW and GYP were adjusted to 140 g/kg grain moisture.

### Statistical Analysis of Phenotypic Data

Thebest linear unbiased estimates (BLUEs) of genetic effects were estimated using the R package *lme4* version 1.1-21 with the following mixed linear model (MLM) (Yang et al., 2017):

$$y_{\mathrm{ijl}}=\mu +g_{i}+e_{l}+ge_{\mathrm{il}}+r_{\mathrm{jl}}+\varepsilon_{\mathrm{ijl}}$$

where *y* *_ijl_* is the phenotypic value of the *i* ^th^ genotype evaluated in the *l* ^th^ environment with the *j* ^th^ replicate, μ is the overall mean, *g* *_i_* is the fixed genetic effect of the *i* ^th^ individual, *e* *_l_* is the fixed effect of the *l* ^th^ environment, *ge* *_il_* is the random interaction effect between the *i* ^th^ individual and the *l* ^th^ environment, *r* *_jl_* is the random effect of the *j* ^th^ replicate within the *l* ^th^ environment, and ε*_ijl_* is the model residuals. The BLUE values of individuals in each experimental population were used as phenotypic data to perform the subsequent analyses, including a GWAS, QTL mapping, and GS.

### Genotyping and Data Analysis

All inbred lines from the natural and biparental populations were used for genotyping, which was performed with the novel developed maize 55 K SNP array (Xu et al., 2017a). As for F_2:3_ population, the DNA extracted from leaves of F_2_ plants was assayed for obtaining genotypic data. Markers with a proportion of missing values >0.10 were removed from the three experimental populations. Finally, a total of 38,299 SNPs with minor allele frequencies (MAFs) > 0.05 were used for further analysis of the natural population. A total of 14,544 and 10,444 SNPs were retained based on chi-square tests (*P* > 0.01) for the RIL and F_2:3_ populations, respectively. The aim of chi-square test is to screen out markers without segregation distortion in biparental populations.

### Genome-Wide Association Study

Marker–trait association mapping was implemented in the R package *GAPIT* version 3.0 with an MLM procedure considering population structure and relative kinship (Q + K model)(Price et al., 2006; Yu et al., 2006; Lipka et al., 2012). PCA was conducted with the *GAPIT.PCA* function in the R package *GAPIT*. The determination of PC number was based on a scree test (Cattell, 1966), and the first seven PCs were selected to construct a covariance matrix to avoid the effect of population structure. A significance threshold of –log_10_ (*P*) > 4 for each trait was employed to identify significant association signals for determining the accuracy and quality of association mapping. The R package *CMplot* version 3.3.3 was used to draw a Manhattan plot (https://github.com/YinLiLin/R-CMplot). The description of candidate genes based on association mapping was based on the maize genetics and genomics database (MaizeGDB, https://www.maizegdb.org/)

### Bin Map Construction and QTL Analysis

The bin maps of the RIL and F_2:3_ populations were aligned and constructed with the sliding-window approach to investigate variant calling errors and calculate the ratio of SNP alleles derived from Zheng58 and HD568. A criterion with a window size of 15 adjacent SNPs and a step size of one SNP was applied to scan the genotypic data. Windows with 11 or more continuous SNPs derived from either parent were regarded as homozygous, and those with fewer SNPs from one parent were recognized as heterozygous. Adjacent windows with the same genotype were combined into one block, and these blocks with different genotypes were inferred to be at or near a recombination breakpoint, allowing bin markers to be designated when consecutive blocks lacked a recombination event across all RILs or F_2:3_ families (Huang et al., 2009; Zhou et al., 2016). A linkage map was constructed using the Kosambi mapping function and the *mstmap* function in the R package *ASMap* version 1.0-4 (Taylor and Butler, 2017). Identification of QTLs for yield-related agronomic traits was performed by the *cim* function in the R package *R/qtl* version 1.44-9 with composite interval mapping (Arends et al., 2010). Then, 1,000 permutation tests with a significance level of *P* < 0.05 were used to determine the threshold likelihood of odds (LOD) ratio for evaluating the significance of each QTL–trait association and for assessing the accuracy and quality of linkage mapping. A 1.5-LOD decrease corresponding to the peak value of the LOD for each bin was defined as the confidence interval for each QTL. Candidate genes identified by linkage mapping were described according to MaizeGDB. In addition, the bin markers were eventually used to perform further GS analysis.

### Genomic Selection

The TRMs detected by association and linkage mapping were used to perform GS for each agronomic trait, and a fivefold cross-validation scheme with 100 replicates was implemented to partition the dataset of each experimental population into training and testing sets and then to calculate the mean correlation coefficient between GEBVs and BLUE values, which represented the prediction accuracy (*r*_MG_). Furthermore, the number of selected markers, including TRMs and randomly selected markers, was set using seven to eight levels (i.e., 20, 50, 100, 500, 1,000, 5,000, 10,000, 30,000, and all markers in the natural population; 20, 50, 100, 500, 1,000, 1,500, 2,000, and all bin markers in the biparental population) to test for a difference in prediction accuracy. As for the TRMs and randomly selected markers, the former was selected according to rank of −log_10_(*P* - value) in association mapping or LOD scores in linkage mapping, the latter was stochastically sampled from whole genome.

### TRM-Based GBLUP Model: Association and Linkage Mapping for the Training Set

Each experimental population was initially partitioned into training and testing sets based on the scheme of fivefold cross-validation, and the training set was used to perform a GWAS or QTL mapping in each cross-validation to identify the TRMs. Subsequently, the GEBVs of individuals in the testing set were estimated by the GBLUP model with different numbers of TRMs, which were compared to the *r*_MG_ based on randomly selected markers to assess prediction accuracy. The GBLUP model was fitted using the R package *BGLR* version 1.0.8 (Pérez and de los Campos, 2014). The hyperparameter settings were based on the default choices in R package, andGibbs sampler was run for 10,000 iterations with the first 5,000 samples discarded as burn in. The general GBLUP model can be described as follows:

$$y=1_{n}\mu +u+\varepsilon$$

where ***y*** is the vector of phenotypic data, **1** *_n_* is the n-dimensional vector of ones, μ is the overall mean, ***u*** is the random effects sampled from the normal distribution *N*(0,***G***$\sigma_{\text{u}}^{2}$), ***G*** is the genomic relationship matrix, and ε is the n-dimensional vector of independent random residuals with the normal distribution *N*(0,***I*** $\sigma_{\varepsilon }^{2}$), in which ***I*** is an identity matrix. The ***G*** matrix was calculated as follows: let ***Z*** = {*z* *_ij_*} be the *n* × *m* matrix of markers, where *n* is the number of individuals in each population, *m* is the number of markers, *z* *_ij_* = 0, 1, or 2 for the *j* ^th^ locus in the *i* ^th^ individual, and *p* *_j_* is the allele frequency of the *j* ^th^ marker. The ***G*** matrix is ***WW***’/2 $\Sigma_{\text{k=1}}^{\text{m}}$*p* *_k_*(1 − *p* *_k_*), where ***W*** = (*w* *_ij_*) with *w* *_ij_* = *z* *_ij_* − 2*p* *_j_* (VanRaden, 2008).

### Extended GBLUP Model Including Additive, Dominance, and Epistatic Effects

The TRMs (mentioned above) were used to fit GS models, and the extended GBLUP model can be described as (Zhao et al., 2015):

$$y=1_{n}\mu +u_{a}+u_{d}+u_{\mathrm{aa}}+\varepsilon$$

where ***u*** **_a_**, ***u*** **_d_**, and ***u*** **_aa_** are the vectors of random effects for additive genetic effects (A), dominance effects (D), and additive-by-additive interaction (AA) effects and are assumed to obey the normal distributions *N*(0,***G*** **_a_**$\sigma_{\text{a}}^{2}$), *N*(0,***G*** **_d_**$\sigma_{\text{d}}^{2}$), and *N*(0,***G*** **_aa_**$\sigma_{\text{aa}}^{2}$), respectively, where ***G*** **_a_**, ***G*** **_d_**, and ***G*** **_aa_** are the genomic relationship matrixes corresponding to additive, dominance, and epistatic genotypic values, respectively. The form of ***G*** **_a_** is identical to that in the general GBLUP model. In addition, the *n* × *m* dominance design matrix ***D*** is defined as follows (Pérez and de los Campos, 2014; Zhao et al., 2015):

$$D=\{d_{\mathrm{ij}}\}=\{\begin{array}{l} -\frac{2p_{12}p_{22}}{\theta },\;\mathrm{if}\;x_{\mathrm{ij}}\;=\;0\\ \frac{4p_{11}p_{22}}{\theta },\;\mathrm{if}\;x_{\mathrm{ij}}\;=\;1\\ -\frac{2p_{11}p_{12}}{\theta },\;\mathrm{if}\;x_{\mathrm{ij}}\;=\;2 \end{array}$$

where *p* *_11_* is the allele frequency of *x* *_ij_* = 0 at the *j* ^th^ locus, *p* *_12_* is the allele frequency of *x* *_ij_* = 1 at the *j* ^th^ locus, *p* *_22_* is the allele frequency of *x* *_ij_* = 2 at the *j* ^th^ locus, and θ = *p* *_11_* + *p* *_22_* − (*p* *_11_* − *p* *_22_*)^2^. Thus, the dominance relationship matrix is ***G*** **_d_** = *n* ***DD***’/trace(***DD***’), where the trace is the sum of all diagonal elements. Then, the epistatic relationship matrix can be calculated by ***G*** **_aa_** = ***G*** **_a_** # ***G*** **_a_**, where the octothorpe denotes the Hadamard product of matrixes.The extended GBLUP models were implemented only for F_2:3_ population. Four models were used to perform GS, namely, the A, A + AA, A + D, and A + D + AA models, and the A model was equivalent to the general GBLUP model. These extended GBLUP models were fitted using the R package *BGLR* version 1.0.8 (Pérez and de los Campos, 2014).

### Fixed-Effects Model Containing the Principal Component Matrix

The TRMs (mentioned above) were used to fit the GS models. A PC matrix was constructed by the *GAPIT.PCA* function in *GAPIT* with TRMs (Lipka et al., 2012) and then added to the BayesC and GBLUP models as a fixed effect. These fixed-effects models were implemented only for natural population. Hence, these models can be described as follows:

y=Xβ+Zu+ε for the BayesC+fixed effects model (BC+PC);

y=Xβ+u+ε for the GBLUP+fixed effects model (G+PC)

where ***X*** is the design matrix for fixed effects consisting of PCs, β is the vector of fixed effect estimates, ***Z*** is the design matrix for random effects in the BC + PC model, and ***u*** is the vector of random effects in both models. Finally, the prediction accuracy assessed by fixed-effect models was compared with the *r*_MG_ estimated by the general GBLUP model with TRMs. The fixed-effects models were fitted using the R package *BGLR* version 1.0.8 (Pérez and de los Campos, 2014).

## Results

### The Quality and Accuracy of Association and Linkage Mapping

The BLUE values of individuals in each experimental population were used to perform association and linkage mapping, and frequency distribution diagrams were drawn (**Supplementary Figure 1**, **2**, and **3**). A total of 11 associated SNPs were identified in the natural population by a GWAS using filtered genotype and phenotypic data, and the number of significant SNPs (i.e., those for which the *P* value surpassed the threshold) for each agronomic trait ranged from 1 to 4 (**Supplementary Figure 4**). For linkage mapping, there were 2,450 and 2,826 recombination bins with an average length of 840 and 727 kb for the RIL and F_2:3_ populations, respectively. Moreover, 79.5 and 86.2% of these bins were <1.0 Mb in segment length for each biparental population. Two high-density genetic maps were constructed using recombination bins as markers based on a chi-square test. The entire genetic distance of each linkage map was 1,811.3 and 1,205.4 cM, and the average and greatest distances between adjacent markers in the respective biparental population were 0.7 and 4.5 cM for the RIL population and 0.4 and 7.8 cM for the F_2:3_ population, respectively (**Table 1**, **Supplementary Figure 5**). With 1,000 permutation tests, the LOD score thresholds for each agronomic trait were ascertained to indicate the presence of a QTL in a particular genomic region (**Supplementary Table 1**). In the RIL population, a total of 16 QTL were identified for six agronomic traits, and the amount of phenotypic variation explained by each QTL ranged from 3.21 to 12.77%. However, a total of eight QTL with negative genetic effects decreased the phenotypic values of agronomic traits when the alleles were identical to the parent conferred a low phenotypic value. In the F_2:3_ population, 38 QTL were detected for the yield-related traits, and each QTL with a LOD value from 5.17 to 26.7 explained 2.49–21.75% of the phenotypic variation. Furthermore, in addition to estimating the additive genetic effects in the F_2:3_ population by QTL mapping, the dominance effects were derived depending on the heterozygous genotypes (**Supplementary Table 2**). In addition, a total of five pleiotropic QTL (pQTL) were detected by integrating the information for 53 QTL obtained from the RIL and F_2:3_ populations, which were distributed on chromosomes 1, 3, 4, 8, and 9 (**Table 2**).

**Table 1:** Summary of the high-density genetic map derived from the RIL and F_2:3_ populations.

Pop.^a^	Chr.^b^	No. of bins^c^	Physical length of map (Mb)	Map length (cM)	Average genetic length (cM)	Maximal genetic length (cM)
RIL	1	436	301.0	309.9	0.7	3.6
	2	301	237.0	240.6	0.8	3.0
	3	292	232.1	224.4	0.8	3.4
	4	233	246.9	156.8	0.7	4.5
	5	298	217.6	276.6	0.9	3.1
	6	202	169.2	128.3	0.6	3.8
	7	204	176.1	141.0	0.7	3.5
	8	209	175.5	150.7	0.7	3.0
	9	132	156.0	93.2	0.7	3.1
	10	143	149.9	89.6	0.6	4.5
	Total	2,450	2,061.3	1,811.3	0.7	4.5
F_2:3_	1	507	301.0	169.3	0.3	1.7
	2	296	237.0	127.7	0.4	3.9
	3	279	232.1	128.1	0.5	3.6
	4	271	246.9	130.8	0.5	7.8
	5	336	217.6	125.3	0.4	2.9
	6	308	169.2	110.8	0.4	2.2
	7	233	176.1	95.4	0.4	3.0
	8	231	175.5	110.2	0.5	2.3
	9	181	156.0	111.6	0.6	7.1
	10	184	149.9	96.0	0.5	2.8
	Total	2,826	2,061.3	1,205.4	0.4	7.8

a Pop.: the experimental populations.

b Chr.: the number of chromosomes.

c No. of bins: the number of bin markers on the chromosome.

**Table 2:** Pleiotropic QTL (pQTL) for each agronomic trait in the biparental populations.

pQTL^a^	Chr.^b^	Interval^c^ (Mb)	Physical length^d^ (Mb)	No. of QTL	Integrated QTL
pQTL1	1	213.1–251.7	38.6	4	*qReh1-2, qRhkw1, qFph1-2, qFhkw1*
pQTL3	3	212.0–216.2	4.2	2	*qFph3, qFeh3*
pQTL4	4	33.7–130.7	97.0	2	*qRel4, qFel4*
pQTL8	8	165.2–168.7	3.5	2	*qRph8, qReh8*
pQTL9	9	17.5–93.3	57.8	6	*qRed9, qRgyp9, qRhkw9, qFph9, qFgyp9, qFhkw9*

a The name of the pleiotropic QTL includes the information of the number of chromosomes.

b Chr.: the number of chromosomes.

c Interval: the confidence interval between two bin markers.

d Physical length: the physical distance between two bin markers based on the B73 genome.

### Effects of Trait-Relevant Markers on Genomic Selection

**Figure 1:**
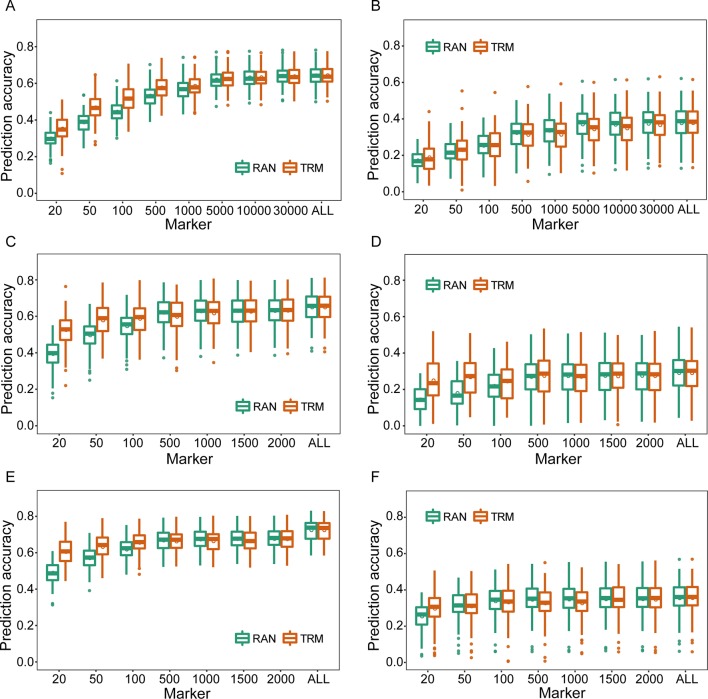
Comparison of prediction accuracies between trait-relevant markers (TRMs) and randomly selected markers based on the results of association and linkage mapping using genotypic and phenotypic data of the training set within the experimental populations. **(A)** and **(B)** Plant height (PH) and grain yield per plant (GYP) in the natural population (*N* = 435); **(C)** and **(D)** PH and GYP in the RIL population (*N* = 212); **(E)** and **(F)** PH and GYP in the F_2:3_ population (*N* = 304). N is the number of individuals in each population. TRM: the prediction accuracy based on TRMs in the general genomic best linear unbiased prediction (GBLUP) model; RAN: the prediction accuracy based on randomly selected markers in the general GBLUP model. ALL: total of 38,299 single nucleotide polymorphisms (SNPs), 2,450 and 2,826 bin markers were used to perform the scheme of cross-validation in natural, recombinant inbred line (RIL), and F_2:3_ populations, respectively. The fivefold cross-validation scheme was implemented in this case.

As for TRM-based GS, the TRMs were initially identified by association and linkage mapping using phenotypic and genotypic data of training set, and the *r*_MG_ based on TRMs was improved compared with that obtained using stochastic markers to perform cross-validation(**Figure 1**). In particular, *r*_MG_ showed significant enhancement when the number of markers was <5,000 in the natural population and 500 in the biparental populations. The degree of improvement in *r*_MG_ estimated by TRMs compared to randomly selected markers for the agronomic traits with low broad-sense heritability was not greater than that for the traits with high broad-sense heritability (the result of broad-sense heritability for each agronomic trait was based on Liu et al., 2018). For instance, the *r*_MG_ based on 20 markers in the RIL population increased from 0.395 to 0.526 for PH and from 0.137 to 0.242 for GYP. In addition, the *r*_MG_ obtained by the TRM-based GBLUP model was the same as that obtained by stochastic markers when the number of markers reached 10,000 for the natural population and 1,000 for the biparental populations (**Figure 1**). In addition, parallel results were obtained for other agronomic traits, including EH, EL, ED, and HKW, when using empirical data to perform cross-validation by TRM-based GBLUP model (**Supplementary Figures 6**–**8**).

### Effects of Nonadditive Effects in the Extended GBLUP Model

**Figure 2:**
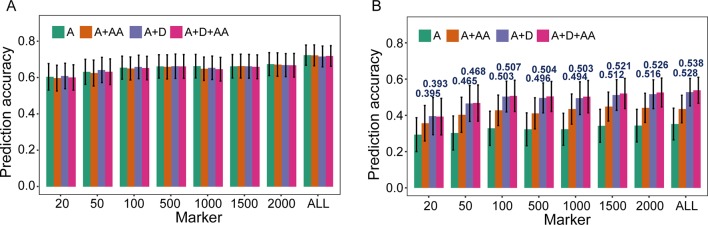
Prediction accuracy of models based on nonadditive effects and trait-relevant markers (TRMs). **(A)** and **(B)** Plant height (PH) and grain yield per plant (GYP) in the F_2:3_ population (*N* = 304). *N* is the number of individuals in each population. The capital letters A, D, and AA refer to additive, dominance, and additive-by-additive interaction effects, respectively. The A model that contains only additive effects is equivalent to the general genomic best linear unbiased prediction (GBLUP) model using trait-relevant markers (TRMs) to perform cross-validation. ALL: total of 2,826 bin markers were used to perform the scheme of cross-validation in F_2:3_ population. The fivefold cross-validation scheme was implemented in this case.

To evaluate the influence of nonadditive effects on *r*_MG_, a total of four statistical models were used to perform fivefold cross-validation in the F_2:3_ population: the A, A + AA, A + D, and A + D + AA models. There two agronomic traits, including PH with high broad-sense heritability and GYP with low broad-sense heritability, were selected to elucidate and demonstrate the utility of including multiple effects in GS models (the broad-sense heritabilityestimated by Liu et al., 2018, 0.83 for PH and 0.65 for GYP). There was no remarkable improvement when integrating dominance and epistatic effects in the GBLUP model, which a target trait with high broad-sense heritability was used for GS (**Figure 2**). However, compared to the GBLUP model that considered only additive effects, the GS model with nonadditive effects significantly enhanced the *r*_MG_ for the traits with low broad-sense heritability. On the other hand, for GYP, statistical models including additive and dominance effects were clearly superior to the additive and epistatic model, and this superiority was consistent across various situations with diverse marker densities. In addition, a slight improvement in *r*_MG_ was observed between the A + D + AA and A + D models in each situation except that in which 20 TRMs were used to fit extended GBLUP models, but the *r*_MG_ was highest for the GBLUP model considering additive, dominance, and epistatic effects. Furthermore, the improvement in *r*_MG_ between the A + D and A + D + AA models became increasingly large as more TRMs were used to construct the genomic relationship matrix. For example, the enhancement of *r*_MG_ increased from 0.003 to 0.010 as the number of TRMs increased from 50 to 2,000 (**Figure 2**). In addition, the proportion of genetic variance explained by dominance effects in the A + D and A + D + AA models was 0.205 and 0.173, respectively. However, the proportion of genetic variance explained by AA interaction effects was 0.127 and 0.032 for the A + AA and A + D + AA models, respectively (**Table 3**).

**Table 3:** Proportions of variance components estimated by the models.

Parameters^a^	PH^b^				GYP			
	A^c^	A + AA	A + D	A + D + AA	A	A + AA	A + D	A + D + AA
$\sigma_{\text{a}}^{2}$	0.564 (0.023)	0.542 (0.023)	0.526 (0.025)	0.510 (0.024)	0.182 (0.014)	0.172 (0.017)	0.171 (0.016)	0.161 (0.017)
$\sigma_{\text{d}}^{2}$			0.043 (0.006)	0.036 (0.004)			0.205 (0.022)	0.173 (0.022)
$\sigma_{\text{aa}}^{2}$		0.032 (0.005)		0.019 (0.003)		0.127 (0.02)		0.032 (0.007)
$\sigma_{\varepsilon }^{2}$	0.436 (0.023)	0.425 (0.024)	0.431 (0.024)	0.435 (0.025)	0.818 (0.014)	0.700 (0.028)	0.624 (0.028)	0.634 (0.026)

a $\sigma_{\text{a}}^{2}$additive genetic variance; $\sigma_{\text{d}}^{2}$:dominance variance; $\sigma_{\text{aa}}^{2}$:additive-by-additive interaction variance; $\sigma_{\varepsilon }^{2}$:estimated error variance.

b PH: plant height; GYP: grain yield per plant.

c The model containing various effects; A, additive genetic effect; D, dominance effect; AA, additive-by-additive interaction effect. The BLUE values of individuals in F_2:3_ population were used as phenotypic data. The extended GBLUP models were implemented for each trait in the F_2:3_ population using 2,000 TRMs. The numbers in the parentheses were standard deviation.

### Using the Information of Population Structure as Fixed Effects to Improve the Predictive Ability of GS Models

**Figure 3:**
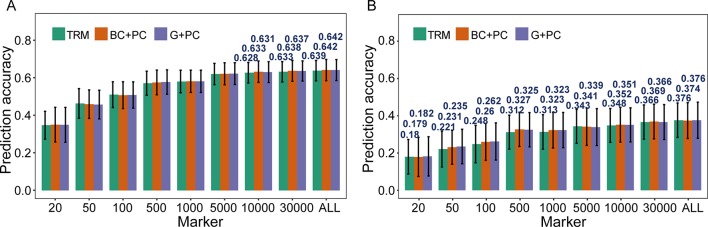
Comparison of prediction accuracy between models based on trait-relevant markers (TRMs). **(A)** and **(B)** Plant height (PH) and grain yield per plant (GYP) in the natural population (*N* = 435). *N* is the number of individuals in each population; trait-relevant marker (TRM): the prediction accuracy based on TRMs in the general genomic best linear unbiased prediction (GBLUP) model; BC + PC: the prediction accuracy based on the BayesC model with PCs as fixed effects using TRMs; G + PC: the prediction accuracy based on the GBLUP model with PCs as fixed effects using TRMs. ALL: total of 38,299 SNPs were used to perform the scheme of cross-validation in natural population. The fivefold cross-validation scheme was implemented in this case.

The possibility of enhancing *r*_MG_ through improved models was investigated using TRMs to construct PC matrix that was appointed as fixed effects and incorporated in models. When PH was used for cross-validation in the case where more than 10,000 TRMs were applied in GS, the *r*_MG_ was slight improvement when PC matrix was included as a fixed effect in the BayesC and GBLUP models (**Figure 3A**). However, for GYP, which had low broad-sense heritability, including population structure information as a fixed effect in GS models enhanced their predictive ability at a moderate marker density. For instance, the *r*_MG_ estimated by 100 TRMs was 0.248 for the general GBLUP model, 0.260 for the BC + PC model, and 0.262 for the G + PC model. Moreover, the maximum degree of improvement in *r*_MG_ was 0.015 when 500 TRMs were used to obtain the PCs for the corresponding design matrix included as a fixed effect in the BC + PC model. The predictive ability of the G + PC model was higher than that of the BC + PC model in some situations when the number of TRMs was <1,000 (**Figure 3B**). However, the superiority of the fixed models to the general GBLUP models with TRMs was small when the number of markers was >5,000.

## Discussion

GS, the theoretical and practical application of marker-assisted selection, has been widely implemented in animal and plant molecular breeding with the accumulation of genotypic and phenotypic data in commercial and experimental breeding programs (Nirea and Meuwissen, 2017; Raoul et al., 2017; Xu et al., 2018; Mastrodomenico et al., 2019; Rezende et al., 2019; Sarinelli et al., 2019; Yuan et al., 2019). Several factors, such as population size, population structure, marker density, heritability, statistical models, and genetic relationships between training and breeding populations, affect prediction accuracy (Schopp et al., 2017; Zhang et al., 2017a; Cerrudo et al., 2018; Edwards et al., 2019; Zhang et al., 2019). Previous researches have been performed using TRMs to test the benefit of employing candidate loci with biological functions based on historical or experimental information (Arruda et al., 2016; Boeven et al., 2016; Rice and Lipka, 2019; Yuan et al., 2019). However, TRMs are usually treated as fixed effects in statistical models. In this study, we aimed to evaluate and discuss the effect of TRMs in various situations using empirical data and proposed statistical models with the purpose of reducing the effect of population structure on prediction accuracy.

Association and linkage mapping are efficient strategies for dissecting the genetic architecture of target traits, and trait-relevant loci can then be used to accelerate the breeding process and assist in improving selection efficiency. According to the results of a GWAS of yield-related traits, two candidate genes related to GYP and HKW were identified by searching the MaizeGDB database, namely, GRMZM2G373928 and GRMZM2G044744, which are located on chromosome 8 (**Supplementary Table 3**). The candidate genes are *ZCN14* and *ZmSSIV*, the first of which is expressed in the tassel, ear primordium, and endosperm and is involved in the early development of kernels (Danilevskaya et al., 2008). The highest expression of the second candidate was detected in the embryo, endosperm, and pericarp 15 days after pollination, and the specific function of this gene is the regulation of starch granule formation, which further affects crop yield and quality (Liu et al., 2015). In addition, the QTL identified by linkage mapping, including *qReh1-2*, *qRhkw1*, *qFgyp5*, *qFhkw3-2*, and*qFhkw7*, are likely important for yield-related traits; the IDs of the corresponding candidate genes are GRMZM2G103773, GRMZM2G018627, GRMZM2G121468, GRMZM5G803935, and AC207722.2_FG009, respectively (**Supplementary Table 3**). In previous studies, these candidate genes were described as having the functions of internode length regulation and photosystem and floral development. The *BRD1* gene associated with EH is essential for internode elongation, and its mutants in maize exhibit severe dwarfism (Makarevitch et al., 2012; Peiffer et al., 2014). The genes *Lhcb2* and *Lhcb9* can encode an apoprotein of light-harvesting chlorophyll-binding protein, which captures light energy for photosystem II (Viret et al., 1993; Boldt and Scandalios, 1995). The *VP15* gene is related to abscisic acid biosynthesis and formation of viviparous seed, and is expressed in both the endosperm and embryo during seed maturation (Suzuki et al., 2006). The gene *TS4* encodes a *mir172* miRNA, which indirectly regulates spikelet meristem determinacy (Xu et al., 2017b). In addition, four QTL with overlapping intervals were detected in the RIL and F_2:3_ populations, namely, *qReh1-2*, *qRhkw1*, *qFph1-2*, and *qFhkw1*, which were linked to QTL affecting PH and yield (**Table 2**). This important region encompasses at least two genes based on the MaizeGDB database, specifically, *BRD1* and *Lhcb9*, implying that pleiotropy or close linkage to other QTL related to various traits exists in this region and further indicating that the genes can be simultaneously inherited by various generations of segregating populations that were constructed by common parents. Moreover, the region might be a hotspot that consists of important QTL with the biological function of controlling PH and kernel weight; understanding the genetic basis of these traits will enable plant breeders to achieve the full yield potential of maize.

GS is the process of using phenotypic and genotypic data from training populations to estimate the GEBVs of individuals in breeding populations based on their genotypic values (Jonas and de Koning, 2013; Desta and Ortiz, 2014; Crossa et al., 2017). Genomic relationships, population structure, and genetic distance can be revealed by genotypic data in combination with statistical and genetic approaches, which will have a crucial impact on the prediction accuracy in GS (Guo et al., 2014; Isidro et al., 2015; Rio et al., 2019). Hence, the number and genetic effects of molecular markers are of great importance in achieving a better *r*_MG_ (Su et al., 2014; Zhang et al., 2014; Ober et al., 2015; Zhang et al., 2015; Arruda et al., 2016; Boeven et al., 2016; Yuan et al., 2019). As for TRM-based GS in this study, the association and linkage mapping were first performed using phenotypic and genotypic data of individuals within the training set; TRMs derived from the training set were used to estimate *r*_MG_. The results in this study illustrate that TRMs can enhance *r*_MG_ in most situations, especially when the marker density within natural and biparental populations is low, as shown by the use of 20–500 functional markers to perform cross-validation and achieve better prediction performance. In this respect, our results are similar to those of several reports that verified the advantage of applying TRMs in GS (Spindel et al., 2016; Yuan et al., 2019). In addition, for biparental populations with comparatively simple population structure and a lower genetic distance between the training and testing sets, the increase in *r*_MG_ is extremely large when a small number of TRMs are used in cross-validation, especially when *r*_MG_ is based on 50 TRMs, in which case, it is approximately equal to the maximum obtained when all randomly selected markers are used to predict the GEBVs of individuals in the testing set. This method can greatly reduce the costs of genotyping in GS-assisted breeding. Regarding the natural population with complex population structure and a greater genetic distance between subgroups, the degree of *r*_MG_ improvement was extremely small when using a few TRMs compared to that obtained with biparental populations. Thus, population structure and genetic relationships may have negative effects on *r*_MG_, and this explanation is supported by previous empirical studies (Guo et al., 2014; Spindel et al., 2015; Rio et al., 2019), which will be further discussed below. On the other hand, it may require extra expenditure when the criteria centered on multiple breeding targets or agronomic traits were used for selection in a breeding program, which the TRMs may vary from one agronomic trait to another. The utilization of overlapped TRMs may be more important and significant in the GS-assisted breeding schemes. In this study, a better prediction performance for different traits were obtained when total of 500–1,000 overlapped TRMs based on the results that all individuals of each population were used to perform association and linkage mapping were applied to implement GS (**Supplementary Figure 9**). Despite that, the application of overlapped TRMs can be limited because the TRMs were different between traits within various experimental populations and agronomic traits had disparate genetic basis and complexity. Hence, the profound study of molecular mechanism of target traits should be required with the purpose of improving the availability and practicability of TRMs in GS breeding programs. In brief, TRMs can be conducive to improving the predictive ability of models and reducing breeding costs for further enhancing genetic gain.

Nonadditive variance, which includes dominance and epistatic effects that generally consist of various interaction effects, such as AA, additive-by-dominance (AD), and dominance-by-dominance (DD) interaction effects, has long been recognized as essential component for dissecting the genetic architecture of target traits and understanding the genetic basis of quantitative traits (Su et al., 2012; Da et al., 2014; Muñoz et al., 2014; Azevedo et al., 2015; Jiang and Reif, 2015; Zhao et al., 2015; Bouvet et al., 2016; Dias et al., 2018; Liu and Chen, 2018; Varona et al., 2018). Several studies were performed using TRMs to test the effect of various combinations of nonadditive effects in extended GBLUP models. For the traits with high heritability, the proportion of nonadditive variance in the phenotypic variance was so small that the degree of *r*_MG_ improvement when the linear mixed model included dominance and epistatic effects had almost no change compared with that obtained with the general GBLUP model considering only additive effects (Wang et al., 2017; Dias et al., 2018; Liu et al., 2018; Alves et al., 2019). For example, PH in the F_2:3_ population has low proportion of nonadditive variance estimated by extended GBLUP models in this study, and then, there was no significant difference between models accounting for various effects, which validates the argument made above. In other words, additive genetic effects can explain the vast majority of genetic variancewhen genomic prediction is implemented for traits with high heritability, which is in agreement with the results of several other studies (Melchinger et al., 2003; Fischer et al., 2008; Hallauer et al., 2010). However, for the traits with high proportion of nonadditive variance, such as GYP in this study, a great improvement in *r*_MG_ was achieved when nonadditive effects were included in statistical models. Furthermore, compared to the additive-effects model, the extent of *r*_MG_ improvement in the A + D model was greater than that in the A + AA model, regardless of how many TRMs were used in cross-validation. This phenomenon may have occurred because considering dominance effects in the predictive model can account for a higher proportion of the genetic variance than including AA interaction effects in the model. Hence, a GS model including additive and dominance effects for a F_2:3_ population with genotypes displaying a high ratio of heterozygous alleles can have important effects on *r*_MG_, and the influence of dominance effects on the enhancement of *r*_MG_ under such conditions can sometimes be superior to that when epistatic effects are considered in GS models. These results were similar to the results of some previous studies (Su et al., 2012; Da et al., 2014; Bouvet et al., 2016; Alves et al., 2019). The largest *r*_MG_ was obtained when the A + D + AA model was implemented in cross-validation, illustrating that fully considering nonadditive effects in extended GS models can further improve predictive performance. However, models including more epistatic effects, such as AA, AD, and DD interaction effects, exhibited a poorer *r*_MG_ than the A + D + AA model and even than the A + D model (results not shown). This phenomenon may result because considering more effects in models can increase their complexity and affect the goodness of fit (Alves et al., 2019). On the other hand, it may be attributed to epistatic effects redundantly involving other effects when various matrixes of genomic relationships are constructed to dissect genetic variance (Hallauer et al., 2010). For instance, additive and dominance effects may be repeatedly considered in other interaction effects, thereby potentially having an undesirable impact on the accuracy of marker effect estimates (Plieschke et al., 2015); however, further study is required to fully reveal the reasons for this finding. In general, to achieve better prediction performance in a heterozygous population, nonadditive effects should be taken into account to enhance predictive ability and accelerate the breeding process.

The formation of population structure and genetic relationships can impact the accuracy of estimates of marker effects in stratified populations and further affect the prediction performance in GS (Technow et al., 2013; Guo et al., 2014; Spindel et al., 2015; Duangjit et al., 2016; Habyarimana, 2016; Rio et al., 2019). There are at least two approaches for reducing the influence of population structure on *r*_MG_. The first is constructing the training population to be closely related to the breeding population before implementing GS (Toosi et al., 2010; Esfandyari et al., 2015; Zhang et al., 2017a). The second is considering the information of population structure as a fixed effect in the model (Guo et al., 2014; Rio et al., 2019). In the case of the former, we investigated the effects of genetic relationships between training and testing sets in a previous publication (Liu et al., 2018). In this study, the effects of population structure on *r*_MG_ were examined using various numbers of TRMs in the models and then discussed. Previous research has explicitly taken genetic structure into account using all markers to fit the modified models without considering TRMs, and *r*_MG_ was not significantly improved compared to that obtained by a general GBLUP model (Rio et al., 2019). The results from this study were similar to those of the abovementioned research when the number of TRMs was >5000 for GYP. In addition, there was a slight improvement in *r*_MG_ when more than 10,000 TRMs were used to perform GS. Therefore, using information of genetic structure estimated with TRMs as a fixed effect can enhance the *r*_MG_ to some extent and may improve the proportion of genetic variance explained by the mixed model. The impact of population structure is attributed to the difference in allele frequencies between groups, which likely affects the estimation of marker effects and cannot be captured by the general parameters of models (Liu et al., 2018; Rio et al., 2019). Hence, developing advanced models that take such information into account will be required to achieve better prediction performance in the future.

## Conclusions

GS has developed with high-throughput genotyping technology and is a landmark for theoretical exploration, from targeting individual loci to considering the whole genome, in the field of animal and plant molecular breeding. Empirical genotypic and phenotypic data from three experimental populations were used to investigate the effects of including TRMs and nonadditive effects in GS models. We found that the *r*_MG_ based on TRMs was better than that obtained by stochastically selected markers, and a few TRMs resulted in a higher *r*_MG_ in biparental populations with simple population structure. In addition, considering nonadditive effects, including dominance, epistatic, and fixed effects, in the statistical models further improved the predictive ability for accelerating the breeding process in cooperation with TRMs. On the other hand, the utilization of TRMs in GS can ensure a sufficient *r*_MG_ for selecting candidate lines and optimize the cost of the breeding cycle, with the strong potential to increase benefits. However, the development of appropriate GS models in the future should take nonadditive effects and information of population structure into account, which can fully capture dominance and epistatic effects on the evaluation of potential hybrids and reduce the effects of population structure, enabling adequate predictive performance when the training and breeding populations are very genetically distant.

## Data Availability

The datasets generated for this study are available on request to the corresponding author.

## Author Contributions

CH and HW conceived and designed the experiments. XL and HW performed the experiments. XL and HW analyzed the data. XH, KL, ZL, and YW contributed materials/analysis tools. XL and HW wrote the paper.

## Funding

This study was supported by the National Key Research and Development Program of China (Grant No. 2017YFD0101201), the Agricultural Science and Technology Innovation Program at CAAS, and the National Basic Research Program of China (973 Program) (Grant No. 2014CB138200).

## Conflict of Interest Statement

The authors declare that the research was conducted in the absence of any commercial or financial relationships that could be construed as a potential conflict of interest.
