# Supplementary material for: Improving Genomic Selection With Quantitative Trait Loci and Nonadditive Effects Revealed by Empirical Evidence in Maize
Source: Front Plant Sci. 2019 Sep 18;10:1129. doi: 10.3389/fpls.2019.01129 (PMC6759780; doi:10.3389/fpls.2019.01129)
Supplement: Supplementary file 1 [file DataSheet_1.docx]

Supplementary Material

**Enhancing genomic selection with quantitative trait loci and non-additive effects revealed by empirical evidence in maize**

**Xiaogang Liu^#^, Hongwu Wang^#^, Xiaojiao Hu, Kun Li, Zhifang Liu, Yujin Wu, Changling Huang^*^**

Institute of Crop Science, National Key Facility of Crop Gene Resources and Genetic Improvement, Chinese Academy of Agricultural Sciences, Beijing 100081, China.

^#^These authors have contributed equally to this work

*** Correspondence:**

Changling Huang

email: [huangchangling@caas.cn](mailto:huangchangling@caas.cn)

## Supplementary Tables and Figures

## 1.1 Supplementary Tables

**Supplementary Table 1.** The threshold of LOD scores for each agronomic trait in RIL and F_2:3_ populations.

| Pop.^a^ | PH^b^ | EH | EL | ED | GYP | HKW |
| --- | --- | --- | --- | --- | --- | --- |
| RIL | 4.67 | 4.51 | 4.59 | 4.45 | 4.66 | 4.44 |
| F_2:3_ | 4.73 | 4.77 | 4.68 | 4.72 | 4.68 | 4.68 |

a Pop.: the experimental populations.

b PH: plant height (cm); EH: ear height (cm); EL: ear length (cm); ED: ear diameter (cm); GYP: grain yield per plant (kg); HKW: hundred-kernel weight (g).

**Supplementary Table** **2.** QTL identified for each agronomic trait in RIL and F_2:3_ populations.

| Pop.^a^ | Trait^b^ | Name^c^ | Chr.^d^ | Pos.^e^ | Flanking markers^f^ | LOD^g^ | PVE^h^ | ADD^i^ | DOM^j^ |
| --- | --- | --- | --- | --- | --- | --- | --- | --- | --- |
| RIL | PH | *qRph2* | 2 | 18.95 | Rmk453-Rmk456 | 5.68 | 4.40 | 3.96 |  |
|  |  | *qRph5* | 5 | 75.47 | Rmk1344-Rmk1346 | 6.37 | 3.21 | 3.36 |  |
|  |  | *qRph7* | 7 | 48.97 | Rmk1833-Rmk1838 | 12.07 | 12.77 | -6.77 |  |
|  |  | *qRph8* | 8 | 128.77 | Rmk2151-Rmk2155 | 7.07 | 9.76 | 5.88 |  |
|  | EH | *qReh1-1* | 1 | 46.45 | Rmk61-Rmk68 | 8.51 | 12.23 | 3.77 |  |
|  |  | *qReh1-2* | 1 | 211.28 | Rmk324-Rmk338 | 4.69 | 11.30 | 3.58 |  |
|  |  | *qReh8* | 8 | 135.67 | Rmk2156-Rmk2164 | 5.36 | 10.70 | 3.51 |  |
|  | EL | *qRel4* | 4 | 39.03 | Rmk1090-Rmk1098 | 8.01 | 7.83 | -4.79 |  |
|  | ED | *qRed4* | 4 | 134.89 | Rmk1244-Rmk1250 | 5.33 | 6.95 | 0.80 |  |
|  |  | *qRed5* | 5 | 36.37 | Rmk1291-Rmk1298 | 8.39 | 10.17 | -0.98 |  |
|  |  | *qRed9* | 9 | 33.94 | Rmk2227-Rmk2240 | 7.82 | 8.49 | -0.89 |  |
|  | GYP | *qRgyp6* | 6 | 126.35 | Rmk1759-Rmk1762 | 6.79 | 9.47 | 0.004 |  |
|  |  | *qRgyp9* | 9 | 33.94 | Rmk2226-Rmk2240 | 4.56 | 6.12 | -0.003 |  |
|  | HKW | *qRhkw1* | 1 | 210.06 | Rmk321-Rmk333 | 7.05 | 11.52 | -1.20 |  |
|  |  | *qRhkw7* | 7 | 116.18 | Rmk1919-Rmk1925 | 7.23 | 11.84 | -1.21 |  |
|  |  | *qRhkw9* | 9 | 45.20 | Rmk2251-Rmk2263 | 5.39 | 8.65 | -1.03 |  |
| F_2:3_ | PH | *qFph1-1* | 1 | 50.11 | Fmk115-Fmk134 | 7.53 | 5.58 | 5.28 | 3.09 |
|  |  | *qFph1-2* | 1 | 108.17 | Fmk337-Fmk347 | 15.19 | 10.34 | 7.99 | 2.15 |
|  |  | *qFph2-1* | 2 | 10.19 | Fmk522-Fmk528 | 6.10 | 2.49 | 3.70 | -1.80 |
|  |  | *qFph2-2* | 2 | 66.93 | Fmk649-Fmk656 | 15.64 | 10.63 | 7.46 | 1.11 |
|  |  | *qFph3* | 3 | 79.52 | Fmk1002-Fmk1007 | 9.10 | 6.83 | -6.33 | 0.49 |
|  |  | *qFph5* | 5 | 31.28 | Fmk1394-Fmk1419 | 5.17 | 4.99 | -2.12 | 7.29 |
|  |  | *qFph7* | 7 | 30.08 | Fmk2082-Fmk2105 | 7.91 | 6.02 | -6.26 | 1.19 |
|  |  | *qFph8* | 8 | 86.25 | Fmk2417-Fmk2429 | 5.88 | 3.59 | 4.70 | 0.06 |
|  |  | *qFph9* | 9 | 41.10 | Fmk2534-Fmk2554 | 6.96 | 2.86 | -3.75 | 2.97 |
|  | EH | *qFeh1-1* | 1 | 65.04 | Fmk170-Fmk187 | 10.36 | 14.18 | 5.42 | 1.91 |
|  |  | *qFeh1-2* | 1 | 113.89 | Fmk357-Fmk369 | 18.27 | 11.73 | 5.25 | 1.22 |
|  |  | *qFeh2* | 2 | 74.79 | Fmk658-Fmk672 | 11.04 | 7.11 | 3.55 | 1.74 |
|  |  | *qFeh3* | 3 | 85.92 | Fmk1006-Fmk1014 | 9.48 | 6.61 | -4.12 | 0.74 |
|  |  | *qFeh8* | 8 | 49.68 | Fmk2312-Fmk2327 | 14.37 | 10.33 | 4.87 | -1.70 |
|  |  | *qFeh9* | 9 | 28.51 | Fmk2503-Fmk2517 | 8.25 | 4.23 | -2.88 | 1.68 |
|  | EL | *qFel2* | 2 | 13.73 | Fmk528-Fmk540 | 5.72 | 4.25 | 3.98 | -0.97 |
|  |  | *qFel3* | 3 | 93.07 | Fmk1018-Fmk1032 | 6.92 | 6.68 | -5.19 | 2.52 |
|  |  | *qFel4* | 4 | 48.92 | Fmk1159-Fmk1177 | 10.84 | 4.77 | -4.29 | 0.83 |
|  |  | *qFel5-1* | 5 | 25.22 | Fmk1394-Fmk1689 | 5.19 | 3.72 | -3.61 | 0.41 |
|  |  | *qFel5-2* | 5 | 124.16 | Fmk1680-Fmk1689 | 5.11 | 3.28 | 3.66 | 0.63 |
|  |  | *qFel8* | 8 | 52.43 | Fmk2319-Fmk2341 | 10.52 | 9.99 | -6.32 | 0.38 |
|  |  | *qFel10* | 10 | 20.24 | Fmk2659-Fmk2665 | 6.31 | 5.58 | -4.73 | 0.47 |
|  | ED | *qFed1-1* | 1 | 56.95 | Fmk148-Fmk161 | 5.41 | 2.91 | -0.62 | 0.37 |
|  |  | *qFed1-2* | 1 | 83.92 | Fmk253-Fmk268 | 8.49 | 6.34 | -0.97 | -0.21 |
|  |  | *qFed2* | 2 | 63.91 | Fmk644-Fmk652 | 9.44 | 19.63 | 1.41 | 0.08 |
|  |  | *qFed5* | 5 | 25.22 | Fmk1393-Fmk1401 | 5.57 | 3.66 | -0.57 | 0.21 |
|  |  | *qFed6* | 6 | 94.64 | Fmk1965-Fmk1974 | 6.93 | 6.27 | 0.77 | 0.10 |
|  |  | *qFed9* | 9 | 57.52 | Fmk2578-Fmk2581 | 8.45 | 7.98 | -0.84 | -0.03 |
|  | GYP | *qFgyp1* | 1 | 110.92 | Fmk154-Fmk364 | 5.62 | 7.00 | 0.005 | 0.01 |
|  |  | *qFgyp5* | 5 | 63.06 | Fmk1515-Fmk1530 | 5.94 | 10.04 | -0.01 | 0.004 |
|  |  | *qFgyp9* | 9 | 39.57 | Fmk2532-Fmk2549 | 5.93 | 8.66 | -0.01 | 0.004 |
|  | HKW | *qFhkw1* | 1 | 105.77 | Fmk327-Fmk344 | 15.62 | 11.81 | -1.26 | 0.12 |
|  |  | *qFhkw3-1* | 3 | 5.80 | Fmk813-Fmk819 | 8.05 | 6.10 | -0.87 | 0.12 |
|  |  | *qFhkw3-2* | 3 | 46.16 | Fmk903-Fmk927 | 6.64 | 2.94 | 0.64 | 0.05 |
|  |  | *qFhkw5* | 5 | 54.44 | Fmk1487-Fmk1513 | 8.90 | 4.95 | 0.71 | 0.54 |
|  |  | *qFhkw7* | 7 | 43.83 | Fmk2121-Fmk2129 | 26.71 | 21.75 | -1.68 | 0.06 |
|  |  | *qFhkw9* | 9 | 41.10 | Fmk2535-Fmk2556 | 8.30 | 5.26 | -0.80 | 0.43 |

^a^ Pop.: the experimental populations.

^b^ PH: plant height (cm); EH: ear height (cm); EL: ear length (cm); ED: ear diameter (cm); GYP: grain yield per plant (kg); HKW: hundred-kernel weight (g).

^c^ Name: the name of each QTL consists of the information of population type (R for RIL population, F for F_2:3_ population), abbreviation of trait, and the number of chromosomes.

^d^ Chr.: the number of chromosomes.

^e^ Pos.: the position of QTL in linkage map.

^f^ Flanking markers: the marginal markers of confidence interval of each QTL.

^g^ LOD: the likelihood of odds.

^h^ PVE: the phenotypic variance explained by individual QTL.

^i^ ADD: the value of additive effect.

^j^ DOM: the value of dominance effect.

**Supplementary Table** **3.** Summary of candidate genes identified by association and linkage mapping.

| Pop.^a^ | Trait^b^ | QTL^c^ | Chr.^d^ | | Pos.^e^ (Mb) | ID of candidate gene^f^ | Function^g^ |
| --- | --- | --- | --- | --- | --- | --- | --- |
| NAT | GYP | *AX.86326709* | | 8 | 25.1 | GRMZM2G373928 | *pebp14* - phosphatidylethanolamine-binding protein |
|  | HKW | *AX.91768443* | | 8 | 124.8 | GRMZM2G044744 | *ss4* - starch synthase4 |
| RIL | EH | *qReh1-2* | | 1 | 250.1 | GRMZM2G103773 | *brd1* - brassinosteroid-deficient dwarf1 |
|  | HKW | *qRhkw1* | | 1 | 246.5 | GRMZM2G018627 | *lhcb9* - light harvesting chlorophyll binding protein9 |
| F_2:3_ | GYP | *qFgyp5* | | 5 | 175.2 | GRMZM2G121468 | *vp15* - viviparous15 |
|  | HKW | *qFhkw3-2* | | 3 | 148.3 | GRMZM5G803935 | *ts4* - tasselseed4 |
|  |  | *qFhkw7* | | 7 | 147.8 | AC207722.2_FG009 | *lhcb2* - light harvesting chlorophyll a/b binding protein2 |

^a^ Pop.: the experimental populations; NAT: natural population.

^b^ EH: ear height (cm); GYP: grain yield per plant (kg); HKW: hundred-kernel weight (g).

^c^ QTL: the name of targeted QTL; The names of SNP markers are regarded as the name of QTL identified by association mapping in natural population; The name of each QTL in biparental populations consists of the information of population type (R for RIL population, F for F_2:3_ population), abbreviation of trait, and the number of chromosomes.

^d^ Chr.: the number of chromosomes.

^e^ Pos.: the physical position of candidate gene based on the B73 genome.

^f^ ID: the ID of candidate gene based on MaizeGDB database.

^g^ Function: the biological function of candidate gene.

## 1.2 Supplementary Figures


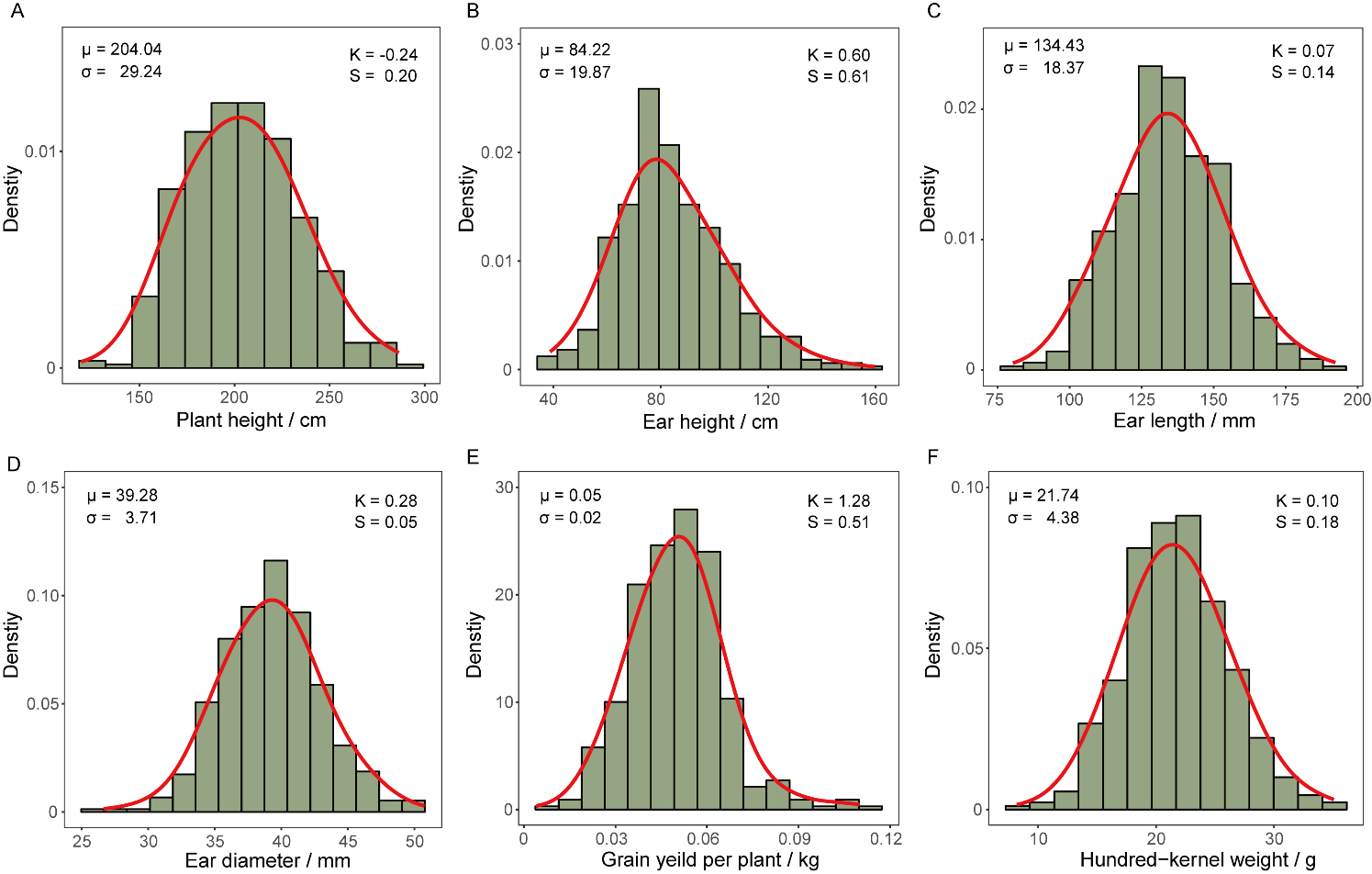


**Supplementary Figure 1.** Frequency distribution diagram of six agronomic traits for BLUE values in natural population. μ: the mean value of agronomic trait; σ: the standard deviation of agronomic trait; K: kurtosis; S: skewness.


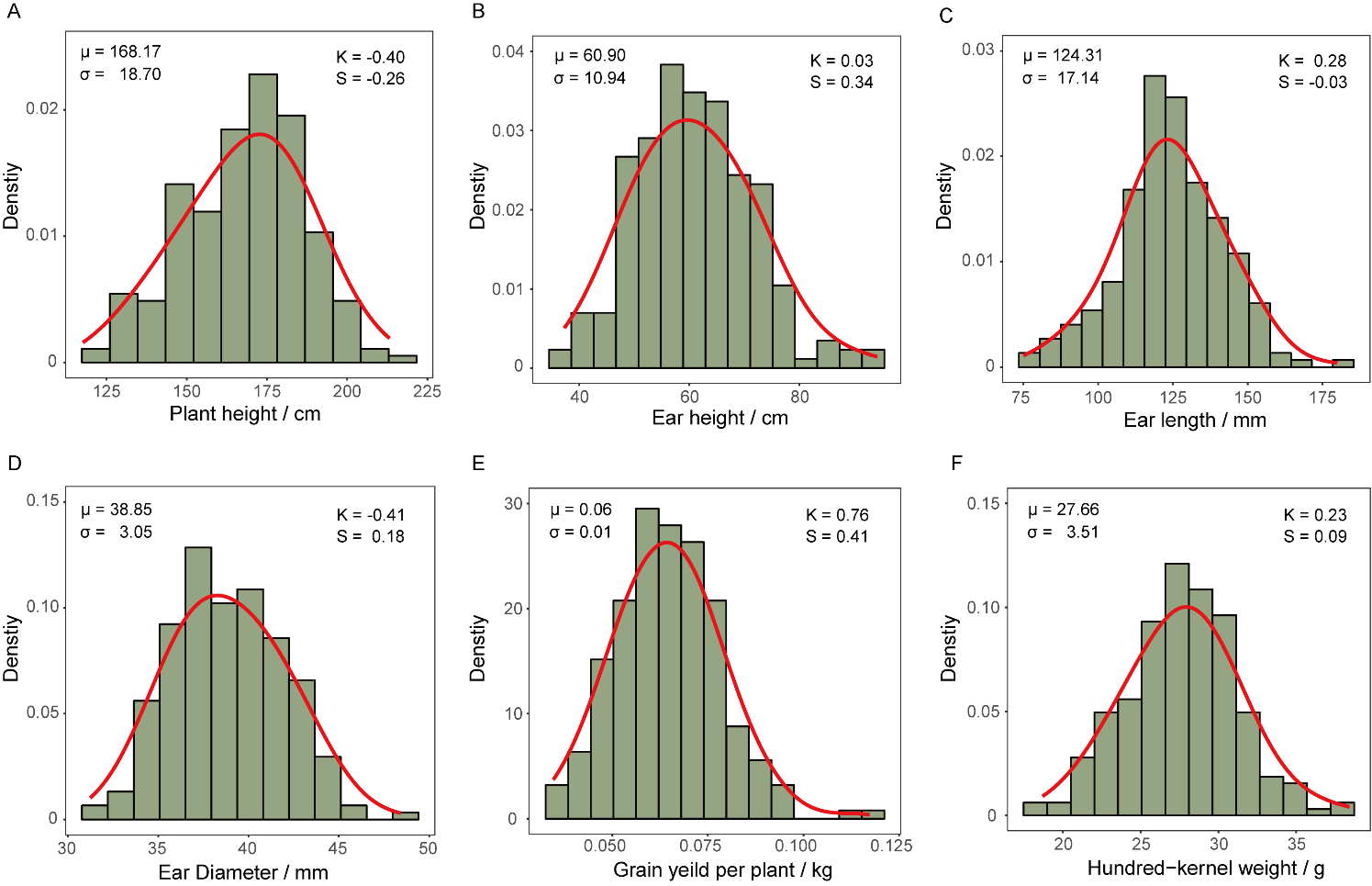


**Supplementary Figure 2.** Frequency distribution diagram of six agronomic traits for BLUE values in RIL population. μ: the mean value of agronomic trait; σ: the standard deviation of agronomic trait; K: kurtosis; S: skewness.


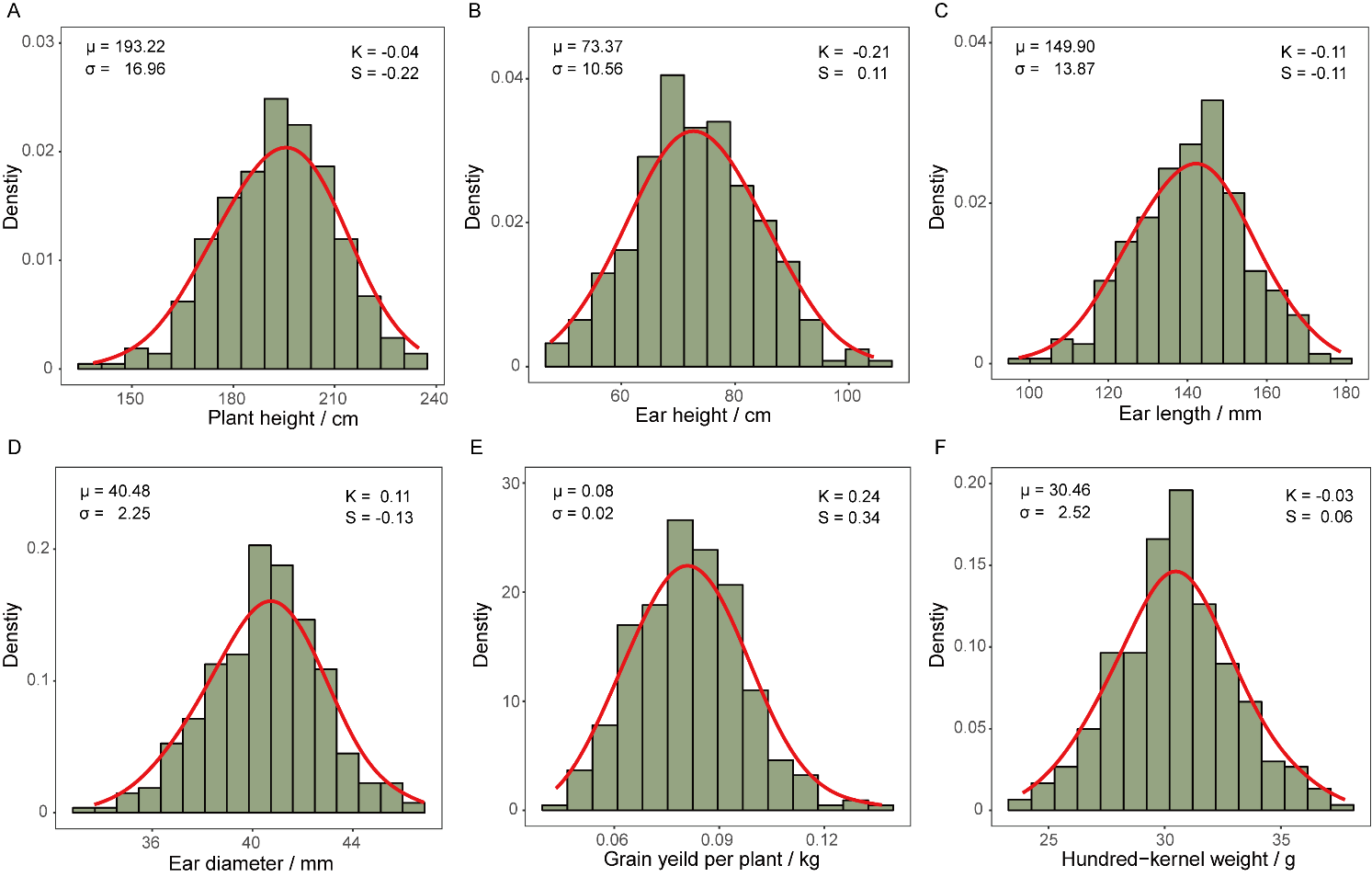


**Supplementary Figure 3.** Frequency distribution diagram of six agronomic traits for BLUE values in F_2:3_ population. μ: the mean value of agronomic trait; σ: the standard deviation of agronomic trait; K: kurtosis; S: skewness.


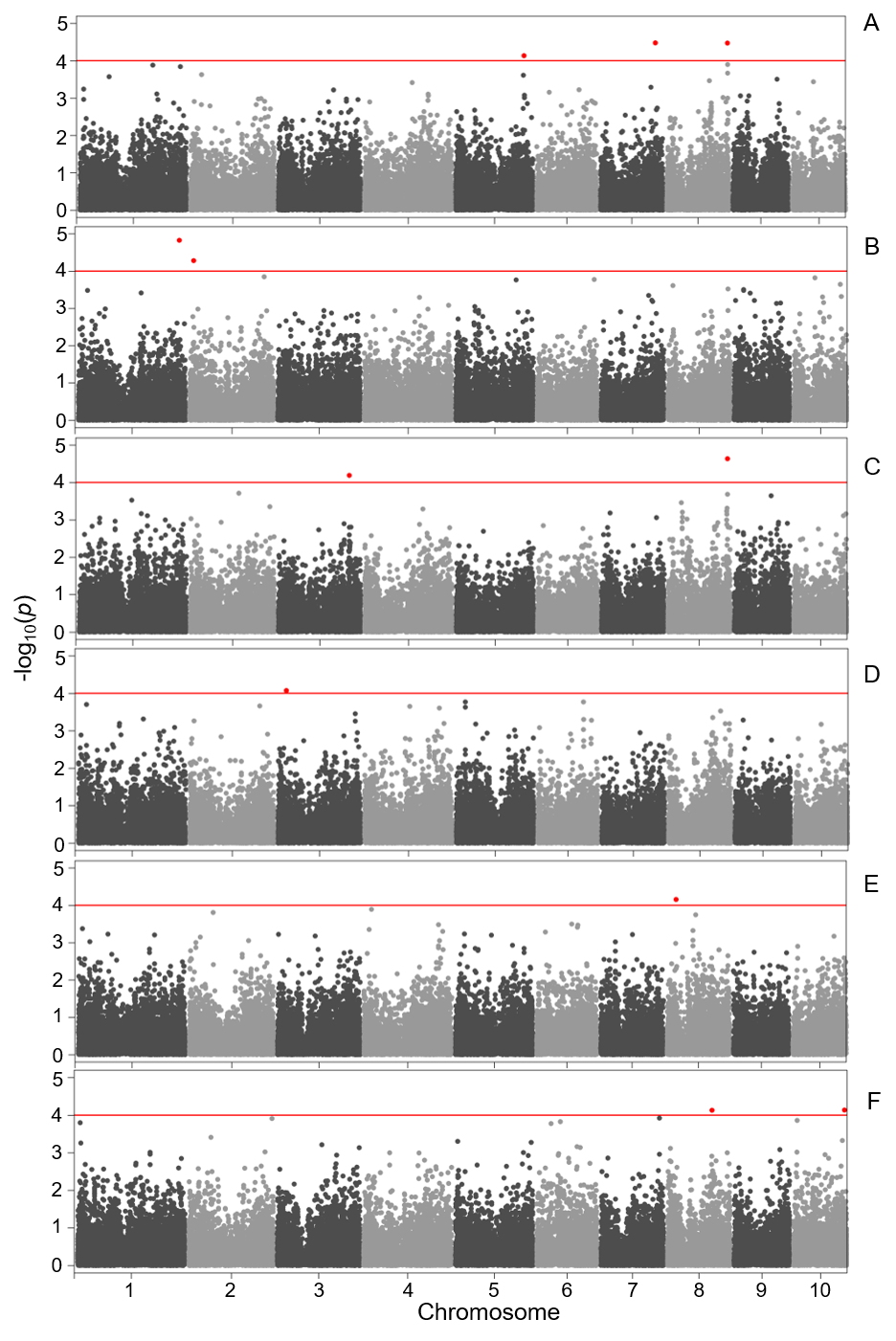


**Supplementary Figure 4.** The Manhattan plots of association mapping for six agronomic traits. (A) to (F): plant height, ear height, ear length, ear diameter, grain yield per plant, and hundred-kernel weight, respectively.


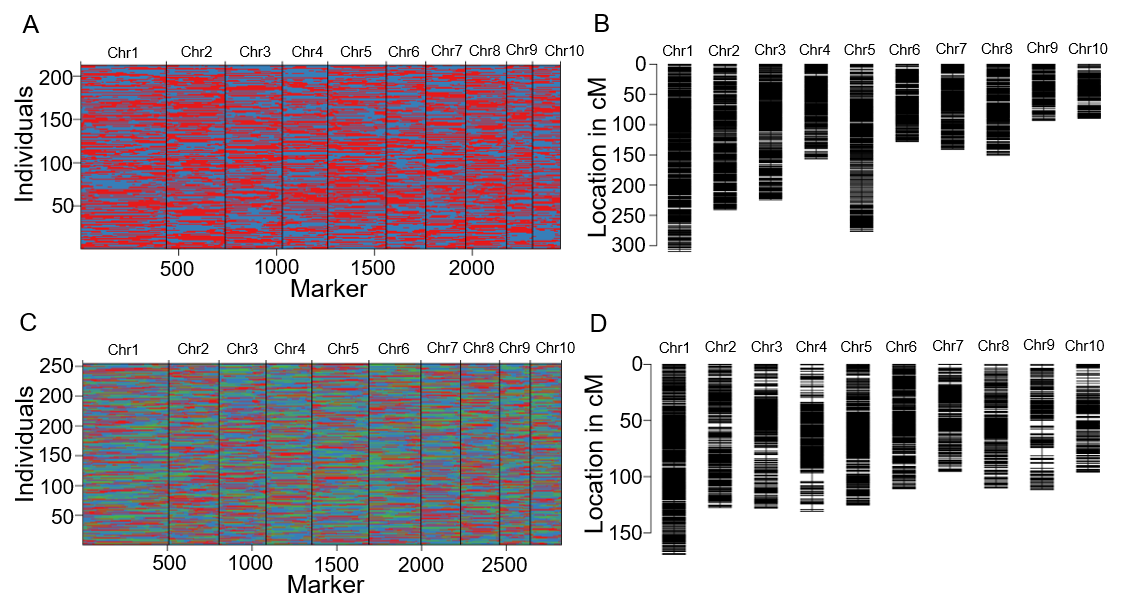


**Supplementary Figure 5.** Recombination bin map and genetic linkage map of RIL and F_2:3_ population derived from Zheng58 and HD568. (A) Recombination bin map of RIL population. Red: the genotype of Zheng58; blue: the genotype of HD568. (B) Genetic linkage map of RIL population. (C) Recombination bin map of F_2:3_ population. Red: the genotype of Zheng58; Blue: the genotype of HD568; Green: heterozygote. (D) Genetic linkage map of F_2:3_ population.


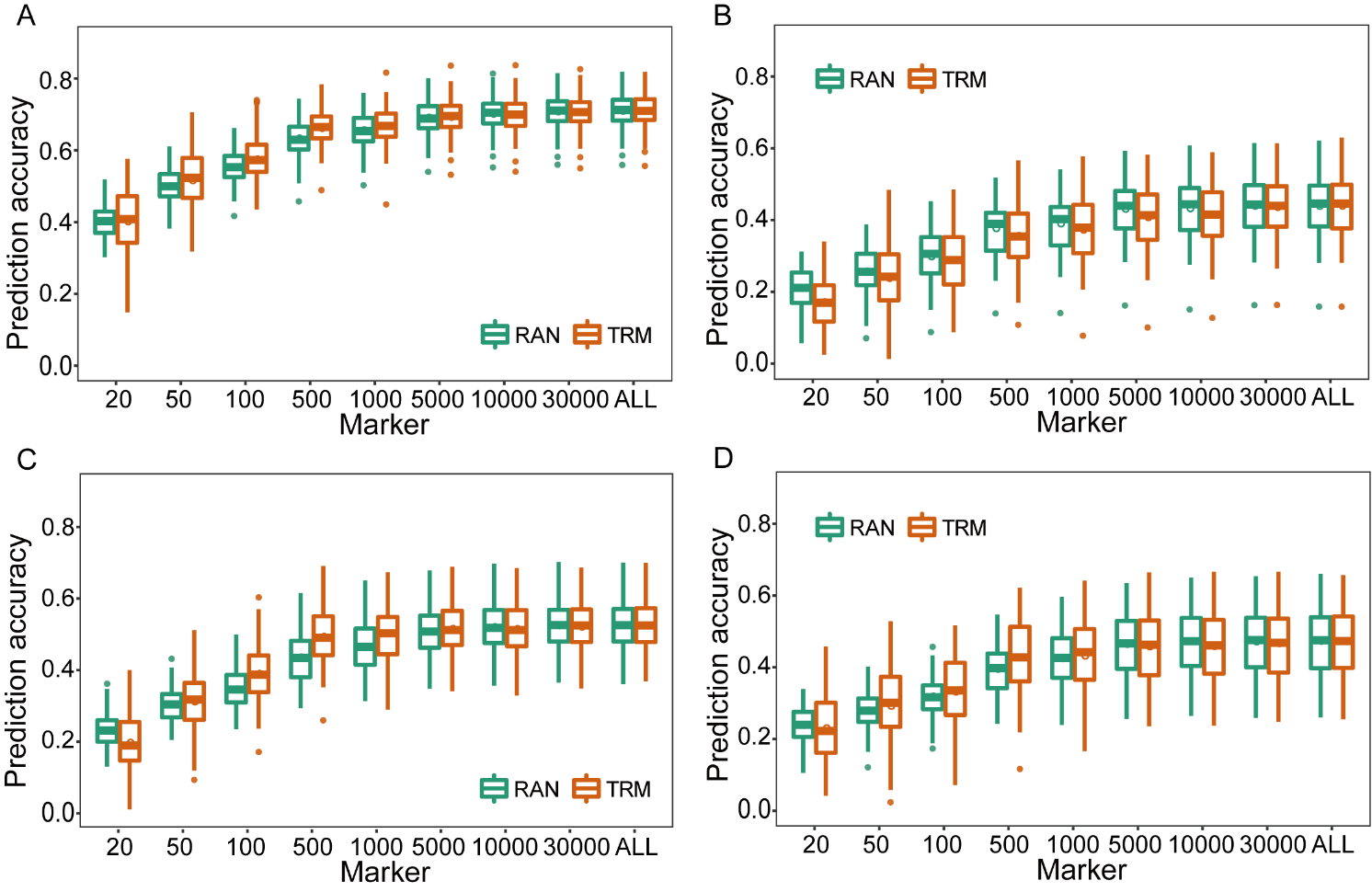


**Supplementary Figure 6.** Comparison of prediction accuracies between TRMs and randomly selected markers based on the results of association and linkage mapping using genotypic and phenotypic data of the training set within natural population (N = 435). (A) to (D) Ear height, ear length, ear diameter, and hundred-kernel weight. N is described as the number of individuals in each population. TRM: the prediction accuracy based on TRMs in general GBLUP model; RAN: the prediction accuracy based on randomly selected markers in general GBLUP model. ALL: total of 38,299 SNPs were used to perform the scheme of cross-validation in natural population. The 5-fold cross-validation scheme was implemented in this case.


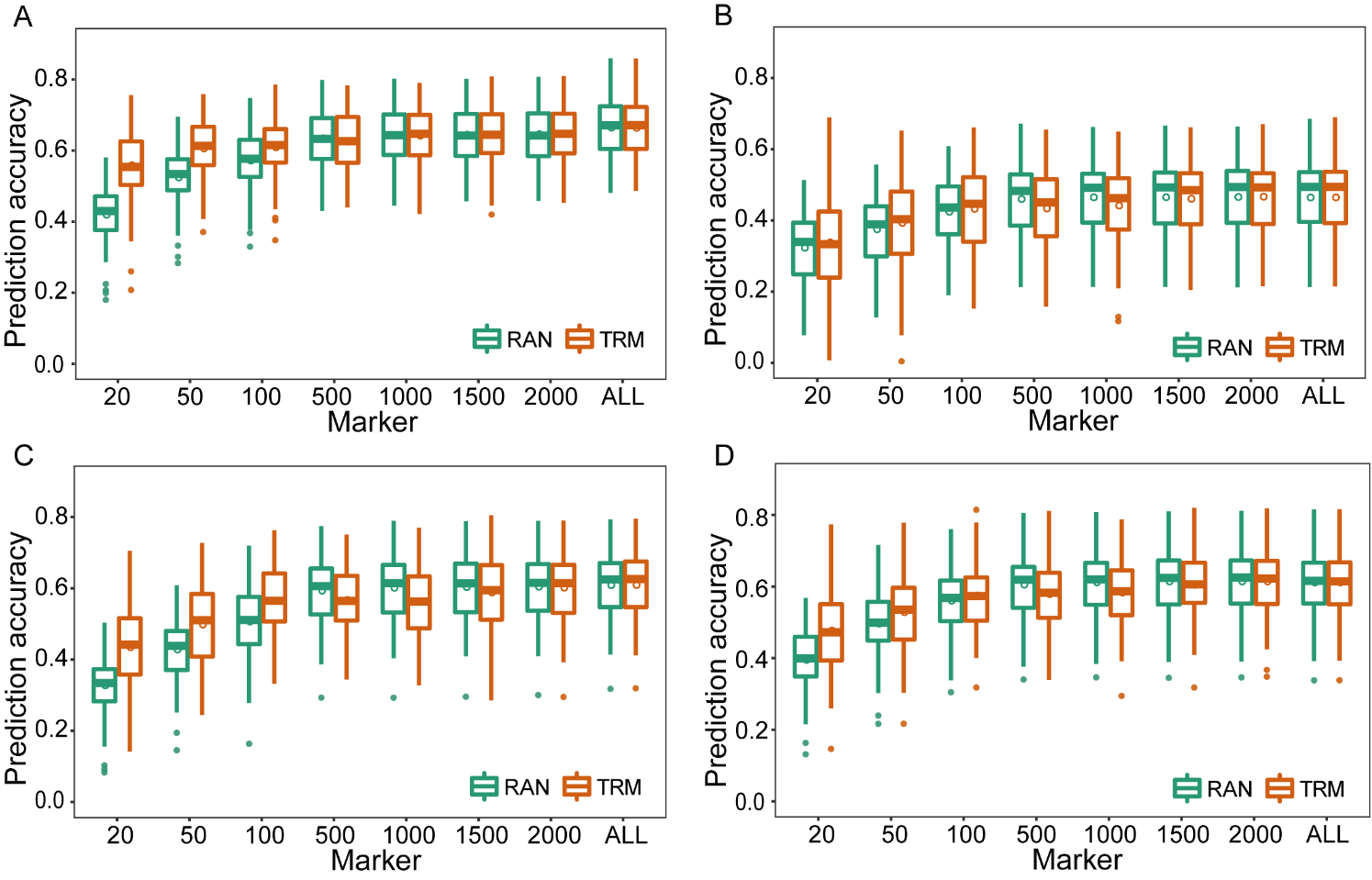


**Supplementary Figure 7.** Comparison of prediction accuracies between TRMs and randomly selected markers based on the results of association and linkage mapping using genotypic and phenotypic data of the training set within RIL population (N = 212). (A) to (D) Ear height, ear length, ear diameter, and hundred-kernel weight. N is described as the number of individuals in each population. TRM: the prediction accuracy based on TRMs in general GBLUP model; RAN: the prediction accuracy based on randomly selected markers in general GBLUP model. ALL: total of 2450 bin markers were used to perform the scheme of cross-validation in RIL population. The 5-fold cross-validation scheme was implemented in this case.


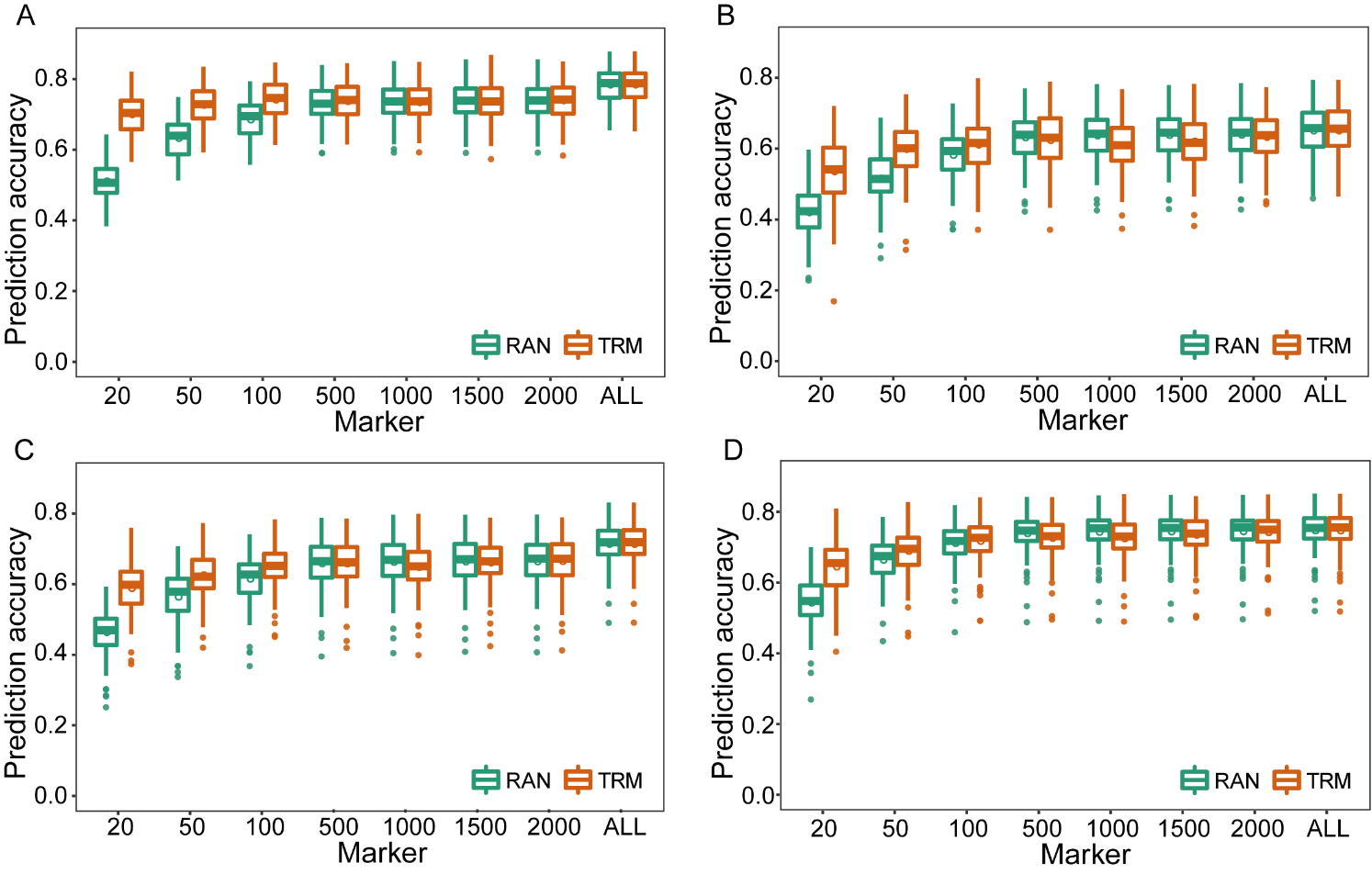


**Supplementary Figure 8.** Comparison of prediction accuracies between TRMs and randomly selected markers based on the results of association and linkage mapping using genotypic and phenotypic data of the training set within F_2:3_ population (N = 304). (A) to (D) Ear height, ear length, ear diameter, and hundred-kernel weight. N is described as the number of individuals in each population. TRM: the prediction accuracy based on TRMs in general GBLUP model; RAN: the prediction accuracy based on randomly selected markers in general GBLUP model. ALL: total of 2826 bin markers were used to perform the scheme of cross-validation in F_2:3_ population. The 5-fold cross-validation scheme was implemented in this case.


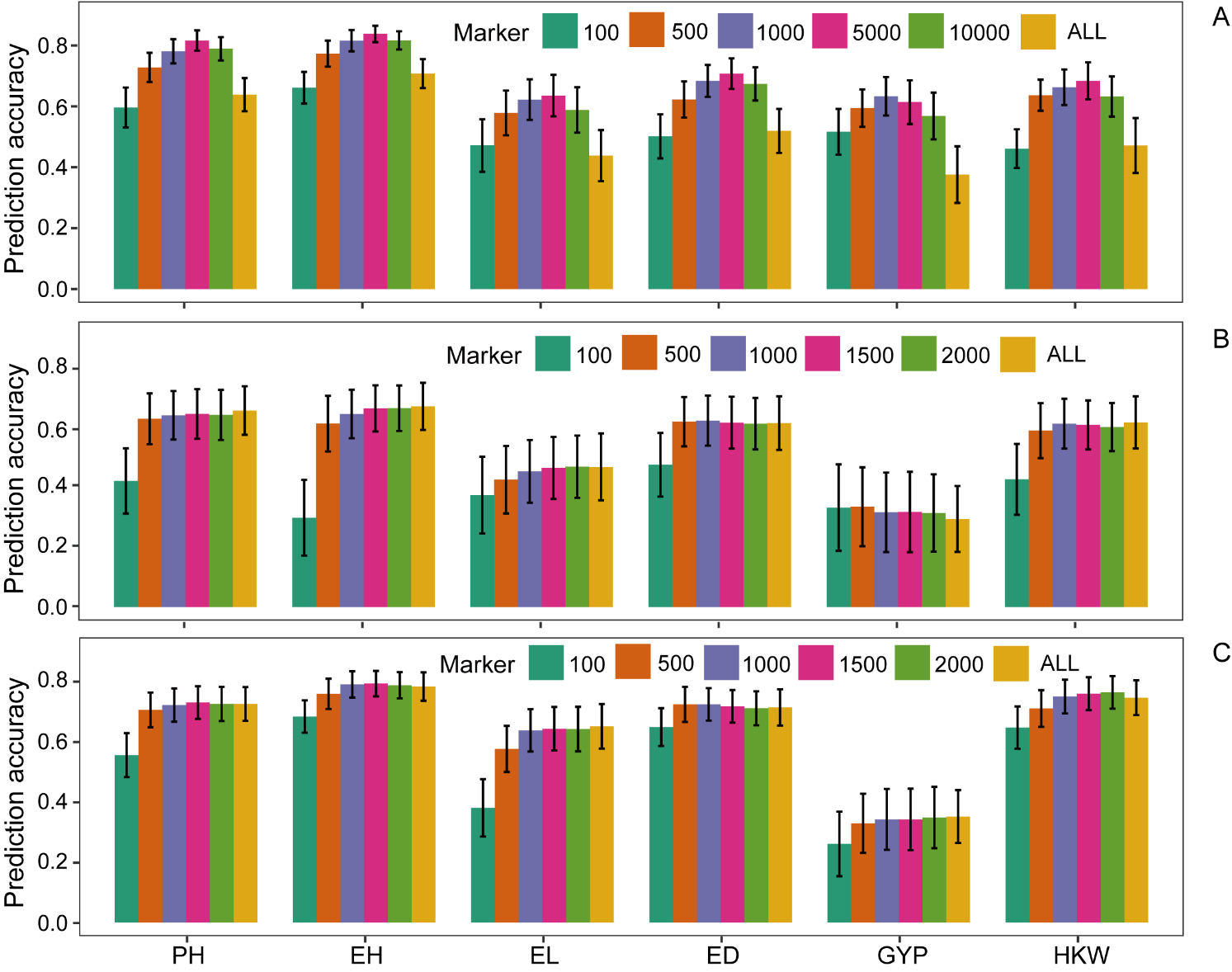


**Supplementary Figure 9.** Comparison of prediction accuracies between overlapped TRMs based on the results of association and linkage mapping using genotypic and phenotypic data of all individuals within three experimental populations. (A) the natural population (N = 435); (B) the RIL population (N = 212); (C) the F_2:3_ population (N = 304). N is the number of individuals in each population. A series of numbers refer to the selection of overlapped TRMs. ALL: total of 38,299 SNPs, 2450 and 2826 bin markers were used to perform the scheme of cross-validation in natural, RIL and F_2:3_ populations, respectively. PH: plant height (cm); EH: ear height (cm); EL: ear length (cm); ED: ear diameter (cm); GYP: grain yield per plant (kg); HKW: hundred-kernel weight (g). The GBLUP model and 5-fold cross-validation scheme were implemented in this case.
